# Blocking activation of the C1r zymogen defines a novel mode of complement inhibition

**DOI:** 10.1016/j.jbc.2025.108301

**Published:** 2025-02-11

**Authors:** Huiquan Duan, Wei Wu, Ping Li, Samuel Bouyain, Brandon L. Garcia, Brian V. Geisbrecht

**Affiliations:** 1 Department of Biochemistry & Molecular Biophysics, Kansas State University, Manhattan, Kansas, USA; 2 Department of Chemistry, Kansas State University, Manhattan, Kansas, USA; 3 Division of Biological and Biomedical Systems, School of Science and Engineering, University of Missouri-Kansas City, Kansas City, Missouri, USA

**Keywords:** complement system, immune evasion, inhibitor, protein structure, zymogen activation

## Abstract

Many hematophagous organisms secrete inhibitors of the coagulation and complement systems as constituents of their salivary fluid. Whereas previous studies on salivary gland extracts from the sandfly *Lutzomyia longipalpis* identified SALO (salivary anticomplement from *L. longipalpis*) as a potent inhibitor of the classical complement pathway (CP), its precise mechanism of action has remained elusive. Here, we show that SALO inhibits the CP by binding selectively to the C1r zymogen. Using surface plasmon resonance, we found that SALO expressed by human embryonic kidney 293(T) cells (eSALO-WT) bound with nanomolar affinity to the zymogen of complement protease C1r (pro-C1r), but that it did not bind the enzymatically active form of C1r. To gain insight into the structural basis for CP inhibition by SALO, we solved a 3.3 Å resolution crystal structure of eSALO-WT bound to a recombinant form of C1r that was engineered to remain in a zymogen-like state (zC1r-12SP). eSALO-WT formed extensive interactions with the zymogen activation loop of zC1r-12SP, including groups derived from residues R463 and I464, which compose its scissile peptide bond. Although the interactions with R463 and I464 were mediated by side-chain sulfation of eSALO-WT at position Y51, we found that this modification enhanced the potency of SALO but was not required for its activity. Consistent with our structural observations, subsequent studies showed that eSALO-WT binding to pro-C1r blocked its activation and thereby inhibited the CP in hemolytic assays of complement function. Together, our results define a new mode of inhibiting complement by blocking the farthest upstream enzymatic reaction of the CP.

The complement system is an ancient arm of innate immunity (1, 2). Although its best understood roles lie in opsonizing and eliminating microorganisms, facilitating clearance of circulating immune complexes, and promoting recruitment of inflammatory and phagocytic cells, contributions of complement to new and unexpected aspects of human physiology continue to emerge (3). Complement activity can be initiated through three conventional routes, known as the classical (CP), lectin, and alternative pathways, each of which responds to specific immunological stimuli. While several immunological triggers of the CP have been described (1, 4), its canonical activator is surface antigen–bound immunoglobulin G (IgG) hexamers. In this scenario, hexamerized IgG molecules serve as an efficient platform for binding the first component of complement (C1) (5).

The C1 complex is a large, ∼766 kDa Ca^2+^-dependent assembly that exhibits specific proteolytic activity upon activation. It is composed of a hexavalent pattern recognition component, known as C1q, bound to a heterotetramer of two serine proteases, known as C1r and C1s (1, 4). Both C1r and C1s are present within resting C1 as zymogens. The earliest events in the CP lead to C1 activation and can be understood as a series of discrete steps (Fig. 1*A*): 1) the C1q component binds specifically to antigen-bound IgGs clustered on surfaces (*e.g.*, microbes), 2) the C1r zymogen undergoes autoactivation into C1r enzyme, and 3) the C1r enzyme cleaves the C1s zymogen into its enzymatically active state. With its C1s constituent now active, C1 can cleave its initial substrate, which is the downstream complement component C4 (C4). This leads to deposition of the C4 fragment, C4b, on complement-reactive surfaces.

**Figure 1:**
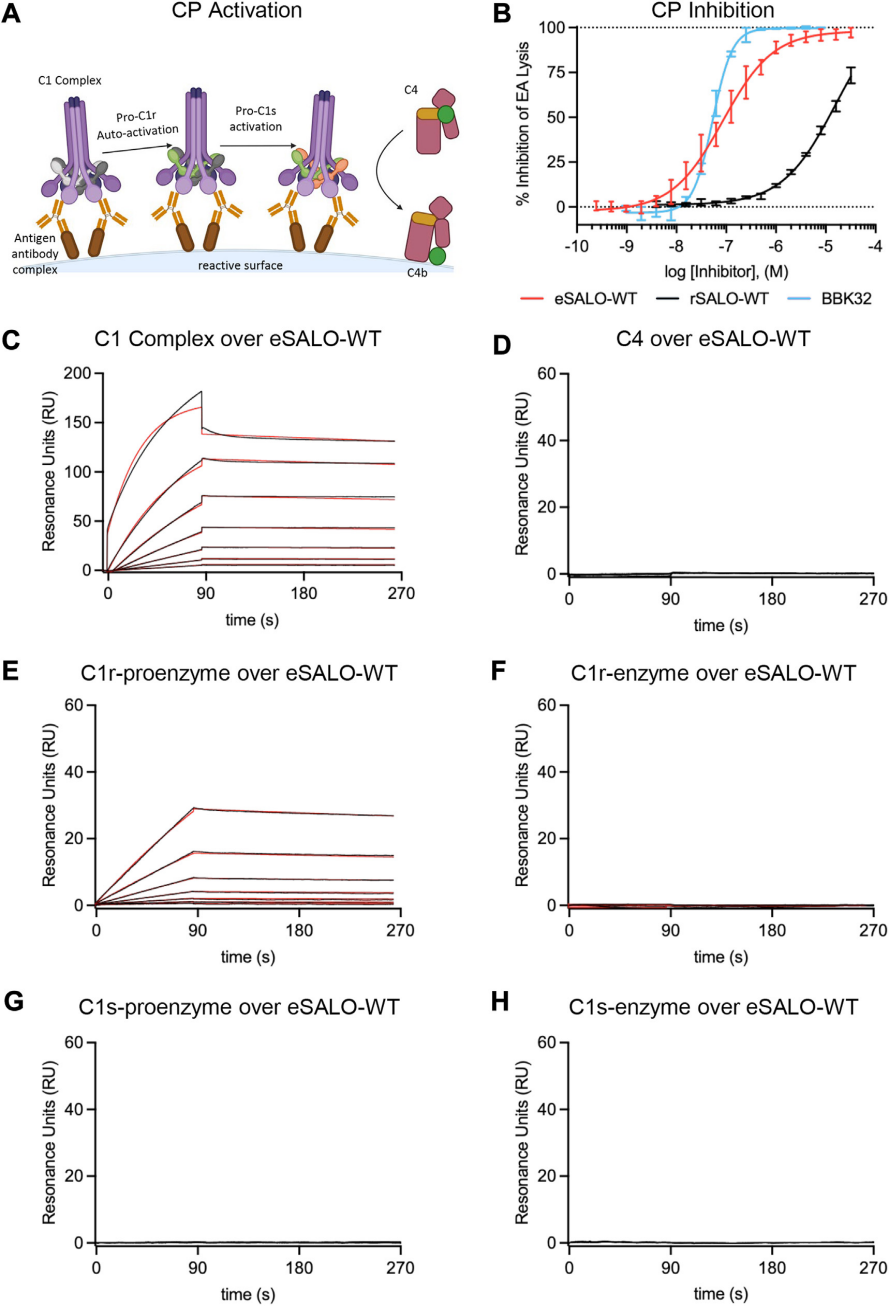
**SALO binds to the C1r zymogen.***A*, schematic representation of the earliest steps in the CP. The C1 complex recognizes antigen-bound antibodies at surfaces, leading to activation of the C1r zymogen. Active C1r converts the C1s zymogen into its enzymatically active form, resulting in cleavage of C4 and deposition of C4b on the surface. *B*, dose-dependent inhibition of the CP by eSALO-WT and rSALO-WT. Antibody-sensitized sheep erythrocytes were incubated with 0.5% (v/v) NHS, resulting in activation of the CP. The extent of complement-dependent lysis was monitored spectrophotometrically and normalized to a control that yielded 100% lysis. The effects of eSALO-WT, rSALO-WT, and the *Borrelia burgdorferi* C1r inhibitor, BBK32 (21), were plotted as a function of concentration prior to curve fitting. Error bars represent the standard deviation from six technical replicates (n = 6). *C*, binding of a two-fold concentration series of C1 to immobilized eSALO-WT. The apparent *K*_*D*_ was 0.8 nM. *D*, binding of a two-fold concentration series of C4 to immobilized eSALO-WT. *E*, binding of a two-fold concentration series of C1r proenzyme to immobilized eSALO-WT. The apparent *K*_*D*_ was 8.5 nM. *F*, binding of a two-fold concentration series of C1r enzyme to immobilized eSALO-WT. *G*, binding of a two-fold concentration series of C1s proenzyme to immobilized eSALO-WT. *H*, binding of a two-fold concentration series of C1s enzyme to immobilized eSALO-WT. Note that in the experiments shown in *D*, *F*, *G*, and *H*, the highest concentration used for each analyte was 1 μM, whereas in *C* and *E*, the highest concentration used for each analyte was 100 nM. The *black lines* represent the reference-subtracted sensorgrams, whereas the *red lines* represent the outcome of fitting to a kinetic model. Representative sensorgrams are shown from a minimum of three technical replicates (n = 3). CP, classical pathway of complement; SALO, salivary anticomplement from *Lutzomyia longipalpis*.

Complement component C2 binds to surface-associated C4b and is also cleaved by active C1. This generates the multisubunit protease known as the CP C3 (complement component C3) convertase (*i.e.*, C4b2a) that activates native C3 into C3a and C3b (1, 4, 6). Surface-bound C3b serves as a platform for assembly of the alternative pathway C3 convertase (*i.e.*, C3bBb) that facilitates self-amplification of the complement response. This same convertase also initiates the terminal complement pathway by cleaving complement component C5 (C5) into C5a and C5b. The terminal pathway activates powerful complement effector mechanisms, including the recruitment of phagocytic cells *via* C5a and formation of the so-called terminal complement complex *via* C5b (1, 2). Thus, a well-orchestrated series of protein–protein interactions and proteolytic transformations underlies initiation of the CP and indeed all complement activities.

*Lutzomyia longipalpis* is a species of sandfly common to Central and South America. *L. longipalpis* serves as a vector for the transmission of *Leishmania* parasites, which pose a significant public health burden in many tropical regions of the world (7). *Leishmania* infection is enabled by the contents of sandfly saliva (8, 9), which contains various anticoagulant and immunomodulatory proteins similar to other hematophagous arthropods including ticks and mosquitos (6, 10). Some of these inhibitors present within insect salivary fluid may also help alleviate gut tissue damage caused by complement components found within the blood meal. In 2016, Ferreira *et al.* (11) reported the identification of one such protein, which they named SALO (salivary anticomplement from *L. longipalpis*). These investigators showed that SALO was a potent dose-dependent inhibitor of the CP that acted upstream of the level of C4 (11). Although they further showed that SALO did not block other complement pathways and that it did not act as a protease inhibitor (11), the mechanistic basis for its activity has remained unclear.

SALO is an ∼11 kDa protein composed of four α-helices (Protein Data Bank [PDB] entry: 5KX4) but bears no sequence relationship to proteins of known function (12). This has largely precluded the use of structural information to formulate hypotheses regarding the mechanism through which SALO inhibits complement. To circumvent this limitation, we employed a biochemical screening strategy in an attempt to decipher the SALO mechanism. We found that SALO inhibits the CP by binding specifically to the zymogen form of C1r. We provide two crystal structure determinations and supporting functional studies which show that SALO binds tightly to the zymogen activation loop of C1r preventing cleavage of its scissile bond. By blocking formation of active C1r, SALO prevents formation of proteolytically active C1. Our results define a novel mode of inhibiting complement by blocking the farthest upstream enzymatic reaction within the CP. They also provide a first-ever description of regulating protease zymogen activation by shielding its scissile bond from proteolytic cleavage.

## Results

### SALO binds selectively to the zymogen form of complement component C1r

Ferreria *et al.* (11) showed that SALO inhibited the CP upstream of the activation of C4. Based upon their observations, we hypothesized that SALO must somehow affect the early part of the CP involving C1 activation (Fig. 1*A*). If this were true, we reasoned that SALO might bind to either C1q, a protease zymogen, or an enzymatically active protease to enable its complement inhibitory activity. To test this hypothesis, we prepared two different versions of recombinant SALO protein (Fig. S1, *A*–*D*). The first version of SALO was expressed as an N-terminal polyhistidine-tagged fusion protein in *Escherichia* *coli* in an insoluble form, purified under denaturing conditions using metal-ion affinity chromatography, refolded by slow dilution of the denaturant, and the monomeric, monodisperse fraction was isolated by size-exclusion chromatography (rSALO-WT). The second form of SALO was expressed as a C-terminal polyhistidine-tagged fusion protein by transiently transfected human embryonic kidney 293(T) (HEK293(T)) cells, secreted into the culture medium, and purified therefrom using a combination of metal ion-affinity and size-exclusion chromatographies (eSALO-WT). We then compared the activity of rSALO-WT to eSALO-WT using a hemolytic assay performed under conditions that stimulate the CP. Curiously, we found that eSALO-WT (IC_50_ = 84.9 nM) was over 100-fold more potent than rSALO-WT (IC_50_ ∼ 16 μM) (Fig. 1*B*). As rSALO-WT was properly folded and maintained some potency in hemolytic assays (Figs. S1*B* & 1*B*), these observations suggested that eSALO-WT might undergo post-translational modification during expression in HEK293(T) cells that contributed to its activity.

We then used surface plasmon resonance (SPR) to screen for interactions between covalently immobilized eSALO-WT with various components of the CP. We found that eSALO-WT displayed dose-dependent binding to intact C1 (Fig. 1*C*). However, eSALO-WT did not bind to C4 (Fig. 1*D*), which serves as the initial substrate for activated C1. As C1 consists of several discrete subcomponents (1, 4), we further screened for interactions between eSALO-WT and both the zymogen and enzymatically active forms of C1r and C1s. Interestingly, we found that eSALO-WT displayed dose-dependent binding to the zymogen form of C1r (Fig. 1*E*), but that eSALO-WT did not bind to enzymatically active C1r (Fig. 1*F*). eSALO-WT also failed to bind either to the zymogen (Fig. 1*G*) or the activated form of C1s (Fig. 1*H*). Thus, these screening data strongly suggested that SALO inhibits the CP by binding to the zymogen form of C1r.

### SALO binds to the activation loop of the C1r zymogen

**Figure 2:**
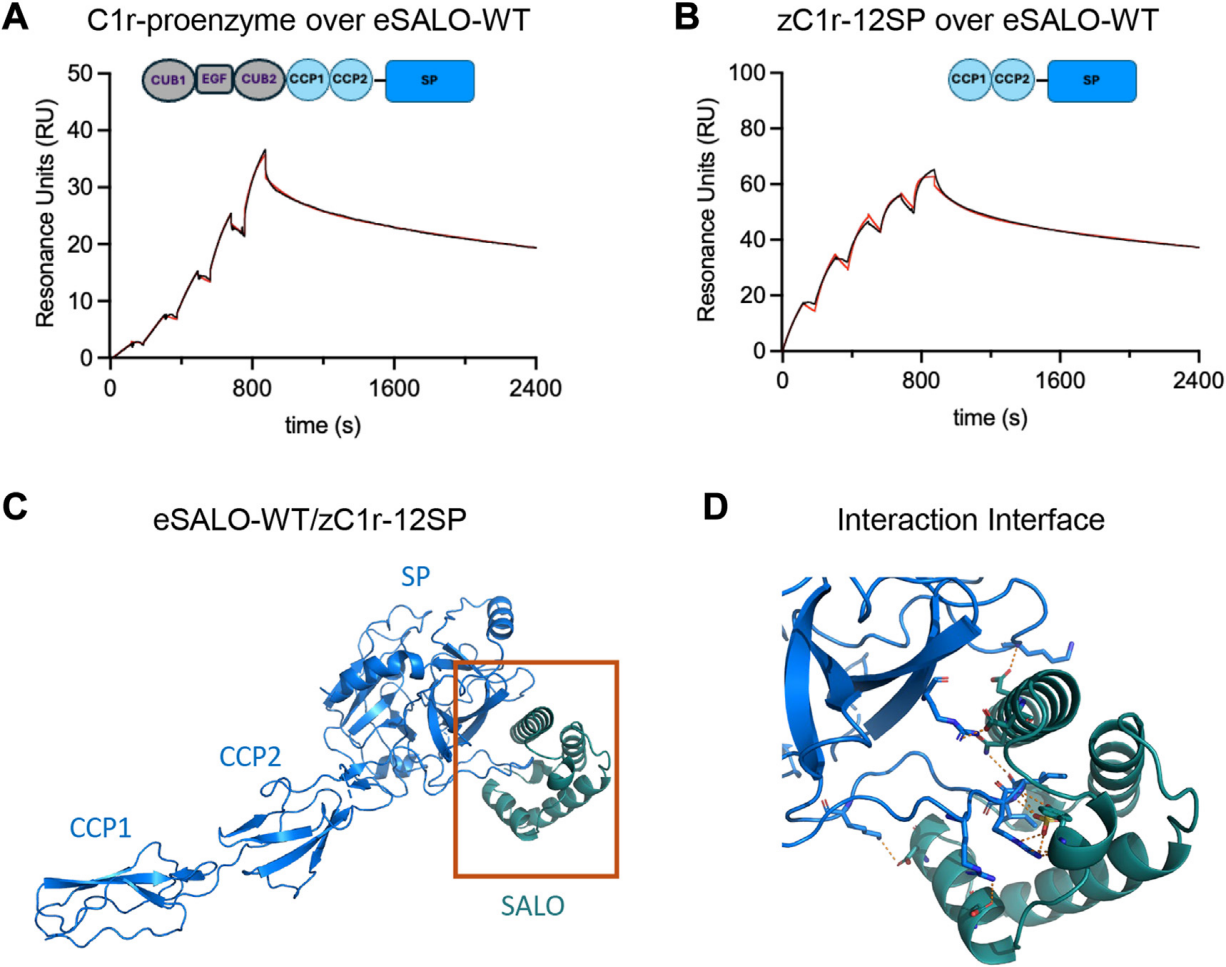
**SALO binds the activation loop of the C1r zymogen.***A*, binding of C1r proenzyme to surface-captured biotinylated eSALO-WT. Interactions were monitored by injecting C1r proenzyme in single-cycle mode, using 25, 50, 100, 200, and 400 nM concentrations of analyte. A representative reference-subtracted sensorgram (*black line*) was analyzed using a two-state kinetic model (*red line*); the apparent *K*_*D*_ was 11.1 nM. A *cartoon diagram* of the domain structure of C1r proenzyme is *inset*. *B*, binding of zC1r-12SP to surface-captured, biotinylated eSALO-WT. Interactions were monitored by injecting zC1r-12SP in single-cycle mode, using 6.25, 12.5, 25, 50, and 100 nM concentrations of analyte. A representative reference-subtracted sensorgram (*black line*) was analyzed using a two-state kinetic model (*red line*); the apparent *K*_*D*_ was 0.7 nM. A *cartoon diagram* of the domain structure of zC1r-12SP is *inset*. *C*, representation of the eSALO-WT–zC1r-12SP structure, determined at 3.3 Å limiting resolution. zC1r-12SP is shown as a *blue ribbon diagram*, whereas eSALO-WT is shown as a *teal ribbon diagram*. Note the only contacts of eSALO-WT with zC1r-12SP involved the SP domain. *D*, close-up view of the interaction interface between eSALO-WT and zC1r-12SP. The region shown in this image corresponds to the area within *orange rectangle* in *C*. SALO, salivary anticomplement from *Lutzomyia longipalpis*.

We sought structural information on the complex formed by SALO and the C1r zymogen. However, since the C1r zymogen undergoes autoactivation over time, we expressed an engineered fragment of human C1r consisting of its two CCP repeats and its serine protease domain, wherein the catalytic serine (S654) had been mutated to alanine (zC1r-12SP). We then used SPR to compare the affinity of surface-captured eSALO-WT for the zymogen form of C1r that had been purified from human serum (Fig. 2*A*) with our zC1r-12SP fragment (Fig. 2*B*). Using a single-cycle kinetics approach, we found that eSALO-WT formed a slowly dissociating complex with both the native and recombinant forms of the C1r zymogen. This was consistent with high-affinity interactions of eSALO-WT for both targets.

Thereafter, we crystallized eSALO-WT bound to zC1r-12SP, collected X-ray diffraction data extending to 3.3 Å limiting resolution, and solved the structure by molecular replacement (Table 1). Following iterative cycles of model building and refinement, our final model had *R*_work_ and *R*_free_ values of 24.2% and 28.7%, respectively (Fig. S2, *A* and *B* & Table 1). The asymmetric unit of the crystal contained two copies of the eSALO-WT–zCr-12SP complex, which superimposed well upon each other (RMSD = 0.34 Å across 444 equivalent Cα positions); thus, our subsequent analyses focused on the eSALO-WT–zC1r-12SP complex represented by chains A and C. Whereas the eSALO-WT–zC1r-12SP interaction buried ∼1100 Å^2^ of surface area at the interface, the most extensive interactions between the two proteins were centered upon the zymogen activation loop of zC1r-12SP (Fig. 2*C*). By contrast, there were no contacts between eSALO-WT and either of the CCP domains of the protease, which may be less accessible within the context of the larger C1 structure (13). Thus, the eSALO-WT–zC1r-12SP complex structure was in good agreement with the pronounced binding selectivity of eSALO-WT for the C1r zymogen when compared with enzymatically active C1r (c.f., Fig. 1*E* *versus* Fig. 1*F*).

**Table 1:** X-ray diffraction data, structure solution, and refinement statistics^a^

PDB accession code	eSALO-WT–zC1r-12SP	eSALO-Y51F–zC1r-12SP
	9EKD	9EKE
Data collection		
Beamline	NSLS-II 19-ID	ALS 5.0.1
Space group	*P*3_2_21	*P*3_2_21
Wavelength (Å)	1.000	0.9744
Cell dimensions		
a, b, c (Å)	117.83, 117.83, 190.58	117.81, 117.81, 195.59
Resolution (Å)	50.00–3.30	50.00–3.10
Wilson *B*-factor (Å^2^)	128.1	75.7
Completeness (%)	100.0 (100.0)	100.0 (100.0)
I/σI	11.5 (1.5)	13.2 (1.0)
R_pim_	0.055 (0.585)	0.062 (0.749)
CC_1/2_	0.930 (0.514)	0.995 (0.441)
Redundancy	17.7 (15.2)	18.1 (16.9)
Refinement		
Resolution (Å)	49.29–3.28	49.36–3.09
Number of reflections	23,486	25,373
*R*_work_/*R*_free_ (%)	24.2/28.7	23.1/27.8
Atoms modeled	7644	7515
Ramachandran plot		
Favored/allowed (%)	89.04/9.46	90.94/7.64
Average *B*-factors (Å^2^)	149.0	91.0
RMSD		
Bond lengths (Å)	0.002	0.002
Bond angles (°)	0.43	0.46

a Values in parentheses are for the highest-resolution shell.

We noted several other interesting features upon closer inspection of the eSALO-WT–zC1r-12SP structure. Foremost, the α-helical bundle fold of eSALO-WT appeared well suited to binding an extended conformation polypeptide (Figs. 2*D*, S3 and S4*A*). eSALO-WT displayed numerous potential hydrogen bond donors and acceptors along the concave channel that is a defining feature of its tertiary structure. Consistent with this, EBI-PISA (14) identified 13 likely hydrogen bonds and four candidate salt bridges between the residues lining this channel, including N33 and Y/X51 (explained further in the following subsection), and residues from the zymogen activation loop of zC1r-12SP, which is composed roughly of residues V458-Q468 inclusive. At the periphery of the eSALO-WT channel, the side chain of E58 formed a salt bridge with the side chain of R461 from zC1r-12SP. It seems that this interaction might help constrain the zymogen activation loop, which constitutes the primary eSALO-WT binding site. Finally, outside the concave channel, residues on the first α helix of eSALO-WT, including D32, appeared to contact a secondary site on zC1r-12SP, which is composed of residues H642–Q647 inclusive. It is possible this secondary site could help contribute to the selectivity of SALO for C1r over other protease zymogens.

### Evidence for sulfation of SALO tyrosine-51

**Figure 3:**
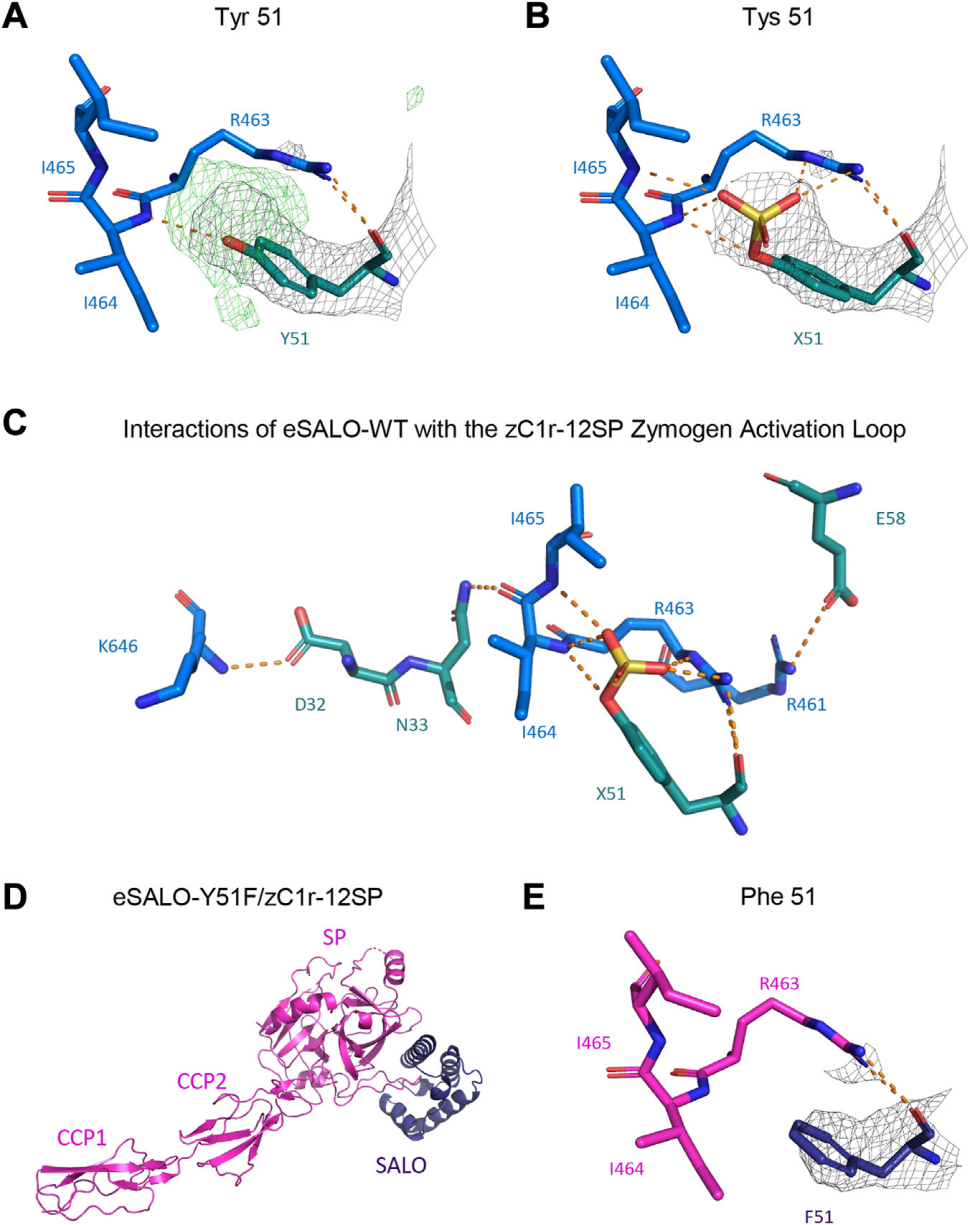
**Sulfation of SALO tyrosine 51 contributes to but is not necessary for binding the C1r zymogen.***A*, close-up view of eSALO-WT Y51 (carbon atoms in *teal*) and its interactions with residues sharing the scissile bond in zC1r-12SP (carbon atoms in *blue*). Likely hydrogen bonds are drawn with *dashed orange lines*. The 2Fo–Fc map (1.2 σ) in the vicinity of Y51 is shown as a *gray mesh*, whereas the Fo–Fc map (3.0 σ) is shown as a *green mesh*. *B*, identical diagram to *A*, except that a tyrosine sulfate (X51) was included in the eSALO-WT model. Note the presence of additional hydrogen bonds formed between X51 and groups from zC1r-12SP. *C*, schematic representation of the interactions found at the interface of eSALO-WT (carbon atoms in *teal*) and zC1r-12SP (carbon atoms in *blue*). The identities of relevant residues are labeled in *teal* for eSALO-WT and *blue* for zC1r-12SP. *D*, representation of the eSALO-Y51F–zC1r-12SP structure, determined at 3.1 Å limiting resolution. zC1r-12SP is shown as a *magenta ribbon diagram*, whereas eSALO-Y51F is shown as a *purple ribbon diagram*. *E*, close-up view of eSALO-Y51F F51 (carbon atoms in *purple*) and its interactions with residues sharing the scissile bond in zC1r-12SP (carbon atoms in *magenta*). Likely hydrogen bonds are drawn with *dashed orange lines*. SALO, salivary anticomplement from *Lutzomyia longipalpis*.

During the course of model building and refinement, we noticed a strong, positive feature in the Fo–Fc electron density map distal to the hydroxyl group of eSALO-WT residue Y51 (Fig. 3*A*). Based on the geometry of this electron density, we suspected that Y51 had undergone post-translational sulfation during eSALO-WT expression and secretion from HEK293(T) cells. We obtained several pieces of analytical information that supported this hypothesis. First, mass spectrometry (MS) analyses on samples of eSALO-WT detected a species whose *m/z* ratio was 80 units greater than expected for unmodified eSALO-WT, which was consistent with the addition of a sulfate group to Y51 (Fig. S1, *C* and *D*). Second, corresponding analyses on samples of a site-directed mutant wherein Y51 was exchanged for F (eSALO-Y51F) did not show a similar species at this increased *m/z* value (Fig. S1, *C* and *D*). Similarly, this increased *m/z* species was not observed in additional site-directed mutants that were prepared for subsequent structure–function studies so long as the Y51F substitution was present (Fig. S1, *C* and *D*). Finally, the presence of tyrosine sulfation could explain the difference in potency between eSALO-WT and rSALO-WT (Fig. 1*B*), since *E. coli* is not known to incorporate this modification into its proteins (15). Therefore, we subsequently modeled this residue as sulfotyrosine, X51 (Fig. 3*B*). In the final model, this sulfate group formed three different hydrogen bonds ranging from 2.6 to 3.2 Å in length with groups derived from R463 and I464, which are the two residues linked by the scissile bond in the C1r zymogen (Fig. 3*C*). It also formed an additional hydrogen bond with the backbone nitrogen from I465, which is immediately C terminal to the C1r zymogen residues that share the scissile bond.

To further investigate this modification, we crystallized the eSALO-Y51F mutant with zC1r-12SP, collected X-ray diffraction data extending to 3.1 Å limiting resolution, and solved the structure by molecular replacement (Table 1). After completing model building and refinement, our final model had *R*_work_ and *R*_free_ values of 23.1% and 27.8%, respectively (Fig. S2, *C* and *D*, and Table 1). We found that the eSALO-Y51F–zC1r-12SP structure largely mirrored that of eSALO-WT–zC1r-12SP (Figs. 3*D* and S2*E*). Although the contributions from the sulfate modification were expectedly absent (Fig. 3*E*), many of the interactions present in the WT structure were maintained. This included the hydrogen bonds formed by D32 and the salt bridges formed by E58 but not the hydrogen bonds formed by N33 (Fig. S4*B*). Overall, these structures showed that sulfation of SALO Y51 promoted interactions with residues sharing the scissile bond of the C1r zymogen, but that sulfation was not necessary for SALO binding to the C1r zymogen.

### Mutagenesis studies identify SALO residues required for binding the C1r zymogen

**Figure 4:**
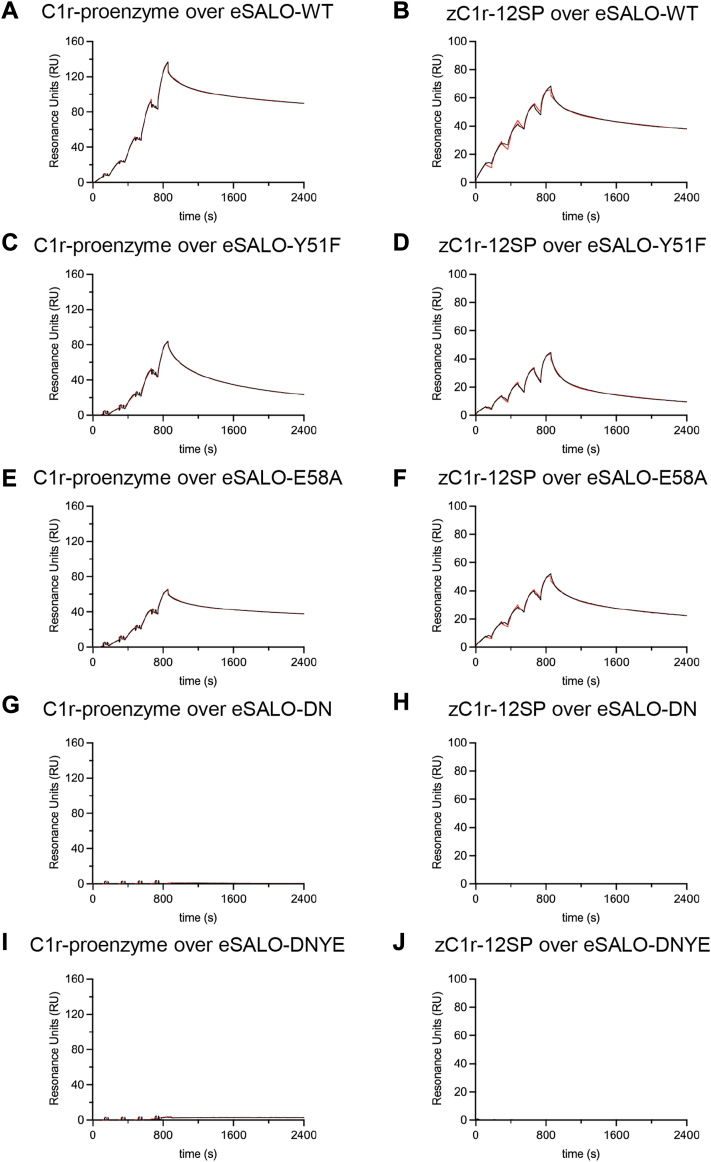
**Characterization of site-directed SALO mutants by SPR.** Binding of C1r proenzyme and zC1r-12SP to surface-captured, biotinylated eSALO proteins was analyzed by SPR. Interactions were monitored by injecting analytes in single-cycle mode, using 25, 50, 100, 200, and 400 nM concentrations of C1r proenzyme and 6.25, 12.5, 25, 50, and 100 nM concentrations of zC1r-12SP for all experiments. Reference-subtracted sensorgrams (*black line*) were analyzed using a two-state kinetic model (*red line*). Representative experiments from three technical replicates (n = 3) are shown, while the final fitting parameters are presented in Table 2. *A*, binding of C1r proenzyme to eSALO-WT. *B*, binding of zC1r-12SP to eSALO-WT. *C*, binding of C1r proenzyme to eSALO-Y51F. *D*, binding of zC1r-12SP to eSALO-Y51F. *E*, binding of C1r proenzyme to eSALO-E58A. *F*, binding of zC1r-12SP to eSALO-E58A. *G*, binding of C1r proenzyme to eSALO-DN. *H*, binding of zC1r-12SP to eSALO-DN. *I*, binding of C1r proenzyme to eSALO-DNYE. *J*, binding of zC1r-12SP to eSALO-DNYE. SALO, salivary anticomplement from *Lutzomyia longipalpis*; SPR, surface plasmon resonance.

We used the crystal structures described previously as a basis for investigating the function of various SALO residues. In addition to eSALO-Y51F, we overexpressed and purified a mutant where E58 was exchanged for alanine (eSALO-E58A) and a double mutant where both D32 and N33 were exchanged for alanine (eSALO-DN); we also prepared a combinatoric mutant where D32, N33, and E58 were exchanged for alanine along with the Y51F substitution (eSALO-DNYE). Each of these eSALO variants was characterized by SDS-PAGE, CD spectropolarimetry, and MS (Fig. S1, *C* and *D*). We then used SPR to examine their affinities for both C1r proenzyme that had been purified from human serum as well as zC1r-12SP (Table 2). Similar to the experiment described previously (Fig. 2, *A* and *B*), we found that eSALO-WT bound with low nanomolar affinity to both analytes as judged by their apparent *K*_*D*_ values of 5.6 ± 1.2 nM and 1.1 ± 0.0 nM, respectively (Fig. 4, *A* and *B*). However, we found that the affinity of eSALO-Y51F for C1r proenzyme and zC1r-12SP was diminished 7.5-fold and 8.2-fold, respectively (Fig. 4, *C* and *D*). Whereas eSALO-E58A displayed only a mild 1.8-fold and 2.3-fold loss of affinity for both analytes (Fig. 4, *E* and *F*), we found that neither eSALO-DN (Fig. 4, *G* and *H*) nor eSALO-DNYE (Fig. 4, *I* and *J*) bound to C1r proenzyme or zC1r-12SP in these experiments.

**Table 2:** Inhibitory potencies^a^ and protein–protein interaction parameters^b^^,^^c^ for various SALO proteins

Analyte	Ligand	IC_50_ (nM)	IC_50_ relative	*K*_*D*_ (nM)	*K*_*D*_ relative	*K*_a-1_ (10^5^ M^−1^s^−1^)	*K*_d-1_ (10^−3^ s^−1^)	*K*_a-2_ (10^−3^ s^−1^)	*K*_d-2_ (10^−4^ s^−1^)
C1r-proenzyme	eSALO-WT	84.9 (75.0–96.1)	1	5.6 ± 1.2	1	0.3 ± 0.1	2.2 ± 0.4	3.0 ± 0.1	2.7 ± 0.1
	eSALO-Y51F	14,520 (10,390–25,410)	171	41.9 ± 22.3	7.5	3.7 ± 4.3	18.3 ± 18.6	0.8 ± 0.7	6.9 ± 3.0
	eSALO-E58A	1197 (1138–1261)	14.1	10.3 ± 2.8	1.8	0.5 ± 0.5	2.7 ± 1.1	2.3 ± 0.3	3.7 ± 0.9
	eSALO-DN	—	—	—	—	—	—	—	—
	eSALO-DNYE	—	—	—	—	—	—	—	—
zC1r-12SP	eSALO-WT	—	—	1.1 ± 0.0	1	3.7 ± 0.1	3.8 ± 0.1	2.4 ± 0.0	2.8 ± 0.0
	eSALO-Y51F	—	—	9.0 ± 1.8	8.2	2.8 ± 0.1	8.0 ± 1.0	1.7 ± 0.0	7.7 ± 0.7
	eSALO-E58A	—	—	2.5 ± 0.1	2.3	2.6 ± 0.1	3.4 ± 0.0	1.8 ± 0.1	4.1 ± 0.1
	eSALO-DN	—	—	—	—	—	—	—	—
	eSALO-DNYE	—	—	—	—	—	—	—	—

a Values in parentheses represent the lower and upper limits for the 95% confidence interval following nonlinear curve fitting.

b All values are presented as the mean ± SD obtained from three replicate injections over the biosensor surface and are taken from the experiments presented in Figure 4.

c Values result from fitting to a two-state binding model.

### SALO inhibits the classical pathway by blocking activation of the C1r zymogen

**Figure 5:**
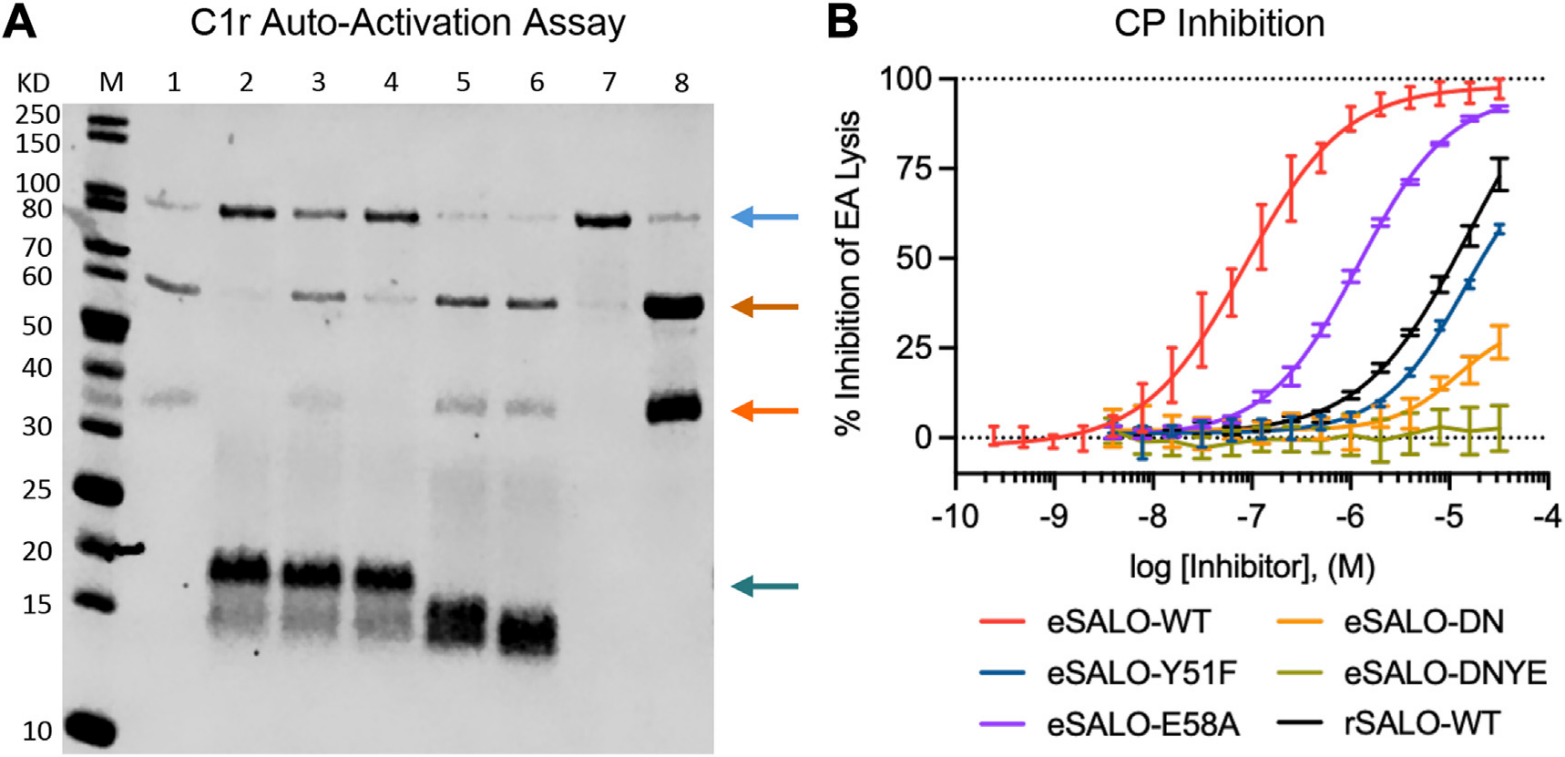
**SALO inhibits the CP by blocking activation of the C1r zymogen.***A*, autoactivation of C1r proenzyme was monitored by SDS-PAGE. Bands corresponding to polypeptides of interest are indicated by *colored arrows*: intact C1r proenzyme (92 kDa; *light blue*); activated C1r, heavy chain (57 kDa; *light orange*); activated C1r, light chain (35 kDa; *dark orange*); eSALO proteins (15–20 kDa; *dark blue*). The identities of individual lanes are as follows: M, molecular weight markers; 1, C1r proenzyme incubated with buffer alone; 2, C1r proenzyme incubated with eSALO-WT; 3, C1r proenzyme incubated with eSALO-Y51F; 4, C1r proenzyme incubated with eSALO-E58A; 5, C1r proenzyme incubated with eSALO-DN; 6, C1r proenzyme incubated with eSALO-DNYE; 7, C1r proenzyme incubated at 4 °C to prevent autoactivation (control); and 8, C1r enzyme (control). The experiment shown is representative of three technical replicates (n = 3). *B*, dose-dependent inhibition of the CP by various SALO proteins. Antibody-sensitized sheep erythrocytes were incubated with 0.5% (v/v) NHS, resulting in activation of the CP. The extent of complement-dependent lysis was monitored spectrophotometrically and normalized to a control that yielded 100% lysis. The effects of various inhibitors were plotted as a function of concentration prior to curve fitting. Error bars represent the standard deviation from six technical replicates (n = 6). CP, classical complement pathway; SALO, salivary anticomplement from *Lutzomyia longipalpis*.

Purified C1r proenzyme is unstable and will undergo spontaneous autoactivation at physiological temperatures. Although this feature reflects the functional role of C1r in complement activation, whether C1r autoactivation occurs in *cis* (16) or in *trans* (17, 18) within the C1 complex remains a topic of investigation. Nevertheless, this process can be monitored by SDS-PAGE as autoactivation of the C1r zymogen results in cleavage of its 92 kDa polypeptide into 57 kDa and 35 kDa fragments. Since our structural observations showed that eSALO-WT binds the zymogen activation loop of zC1r-12SP (Figs. 2 and 3), we hypothesized that eSALO-WT binding would prevent C1r zymogen autoactivation. To test this idea, we incubated C1r proenzyme with either eSALO-WT, the mutant forms of eSALO described previously, or buffer alone at 37 °C for 10 h. We then separated the reaction products by SDS-PAGE and visualized the contents by Coomassie Brilliant Blue staining (Fig. 5*A*). While C1r zymogen underwent efficient autoactivation in this experiment, we found that eSALO-WT blocked the autoactivation reaction. Moreover, we noticed that the ability of the eSALO variants to block autoactivation correlated with their affinity for C1r proenzyme as measured by SPR (Table 2). eSALO-E58A was less effective than eSALO-WT but was better than eSALO-Y51F in blocking C1r autoactivation. Both eSALO-DN and eSALO-DNYE failed to block C1r autoactivation altogether. Thus, SALO binding was required to block autoactivation of the C1r zymogen.

We previously found that eSALO-WT blocked CP-driven hemolysis with an IC_50_ value of 84.9 nM (Fig. 1*B*). Therefore, we used identical hemolytic assays to test if the apparent IC_50_ values of these eSALO variants correlated with their ability to bind C1r proenzyme and block its autoactivation (Fig. 5*B* & Table 2). We found that eSALO-E58A displayed an IC_50_ value diminished by 14.1-fold, whereas eSALO-Y51F was even less potent with an estimated 171-fold loss compared with eSALO-WT. We noted that rSALO-WT was of similar potency to eSALO-Y51F, which suggested that the differences between these two variants and eSALO-WT were largely because of sulfation of Y51. Finally, both eSALO-DN and eSALO-DNYE had little inhibitory activity, which was consistent with their behavior in the autoactivation assay, as well as in direct binding studies. Therefore, SALO binding to the C1r zymogen blocks formation of active C1r enzyme, which results in potent inhibition of the CP. Since activation of the C1r zymogen is the furthest upstream proteolytic event in the CP, our observations here define a novel mode of complement inhibition.

## Discussion

Although SALO was identified as a potent, CP-specific inhibitor nearly a decade ago (11), the molecular basis of its mechanism has remained unclear since that time. Here, we used a biochemical screening strategy to show that SALO binds selectively and with nanomolar affinity to the zymogen of C1r (Fig. 1). Our structural studies revealed that SALO binds to the activation loop of the C1r zymogen (Figs. 2 and 3; S2 and S3), shielding its scissile bond from proteolytic cleavage (Fig. 5). While we determined that the interaction between SALO and C1r proenzyme is enhanced by sulfation of SALO Y51 (Figs. 3, S1, S3 and S4), we also found that this modification is not required for SALO binding or activity (Figs. 4 and 5). By blocking the first enzymatic step in C1 activation, SALO affects robust inhibition of the CP (Fig. 5). To our knowledge, this sort of mechanism defined by SALO has not been described previously. Therefore, it constitutes a novel mode of complement inhibition not seen in other naturally occurring regulatory molecules, immune evasion proteins, or therapeutic modalities.

During the course of this study, we determined that SALO expressed from HEK293(T) cells was modified by tyrosine sulfation at position Y51 (Figs. 3, S1, S3 and S4). Although this finding was unanticipated, the presence of positive Fo–Fc electron density overlapping the phenolic oxygen of Y51 in the eSALO-WT–zC1r-12SP structure strongly suggested this possibility. We attempted to confirm the presence of tyrosine sulfation in eSALO-WT samples using immunoblotting but were unsuccessful with that approach. However, using high accuracy MS, we detected species of 80 *m/z* greater than expected in samples of eSALO-WT, eSALO-E58A, and eSALO-DN but not in samples of eSALO-Y51F or eSALO-DNYE, where the modification site (*i.e.*, Y51) had been mutated (Fig. S1); this *m/z* change corresponded very well to the increased molecular weight expected from addition of a sulfate group. Since the MS data we obtained are not quantitative, it is not possible to know the extent of tyrosine sulfation in these samples. Nevertheless, incorporation of tyrosine sulfate (*i.e.*, X51) instead of tyrosine alone (*i.e.*, Y51) in the eSALO-WT–zC1r-12SP structure improved the model to electron density map correlation and appeared to result in the formation of four additional hydrogen bonds, three of which involved R463 and I464, which are the residues that share the scissile bond in the C1r zymogen (Figs. 3 & S4). At the functional level, this modification appeared to result in an ∼8-fold gain in affinity for the C1r zymogen and an ∼170-fold gain in potency in CP-dependent hemolytic assays, as judged by comparison of eSALO-WT and eSALO-Y51F (Figs. 4 and 5, and Table 2). The existence of two likely tyrosine protein sulfotransferase isoforms in *L. longipalpis* that share 78% and 83% identity to the *Drosophila melanogaster* TPST, Tango-13, strongly suggests that natively expressed SALO will undergo tyrosine sulfation to some extent. Conceivably, this could explain the potent CP inhibitory activity ascribed to *L. longipalpis* salivary gland homogenates some years ago (19).

Given the complex structure–function relationships of C1 and its essential role in triggering CP activity (Fig. 1*A*), it is not surprising that several different immune evasion proteins acting at the level of C1 have been described (6). Perhaps the best understood among these come from the Lyme disease spirochete, *Borrelia burgdorferi*, which expresses at least two different classes of C1 inhibitors (20). The cell-surface lipoprotein BBK32 binds to the active site of C1r enzyme, thereby blocking not only C1r autoactivation but activation of the C1s zymogen as well (21, 22, 23). It also binds tightly to the zymogen of C1r, albeit through a distinct binding site from the activation loop recognized by SALO (Figs. 2 & S2; (23)). Separately, the *B. burgdorferi* outer membrane lipoproteins ElpB and ElpQ both bind to the activated forms of C1r and C1s (20). Although no crystal structures for their representative complexes with either protease are available, biochemical studies have shown that both ElpB and ElpQ interfere with recognition of the substrate, C4, without occluding the C1s active site (24, 25). Despite this elaborate and seemingly redundant complement evasion program, no protein with a mechanism comparable to SALO has been identified from *B. burgdorferi* or related spirochetes (26). While this does not rule out the existence of such a molecule, the divergent mechanisms displayed by SALO *versus* BBK32, ElpB, and ElpQ could reflect evolutionary adaptations to different selective pressures faced by hematophagous arthropods like *L. longipalpis versus* bloodborne bacteria like *B. burgdorferi* (6). They may also underscore unique requirements for effectively inhibiting C1 in solution as opposed to on surfaces. Exploring this latter possibility further will be a topic for future investigations.

Several C1 inhibitors have either been approved for therapeutic use in humans or are undergoing investigation for treating CP-linked conditions, including hereditary angioedema, cold agglutinin disease, and immune thrombocytopenia, among others (reviewed in Refs. (27, 28, 29)). The most established of these is the aptly named C1 inhibitor (C1-INH), which is marketed under various trade names depending on the manufacturer for treatment of hereditary angioedema. Forms of C1-INH are also being evaluated for treating ischemia–reperfusion injuries and organ transplantation in phase II and phase III clinical trials. C1-INH is a SERPIN and inhibits the active forms of both C1r and C1s. However, it also inhibits activated mannose-binding lectin-associated serine proteases that function within the lectin pathway, as well as several activated coagulation factors, and is therefore not specific in its action against the CP. By contrast, the anti-C1s monoclonal antibody, sutimlimab, specifically blocks cleavage of the substrates, C4 and C2, in response to CP activation. Sutimlimab was recently approved for the treatment of cold agglutinin disease (30), providing transformative evidence that an individual subcomponent of C1 could be selectively targeted for therapeutic purposes. In that regard, we have shown that SALO inhibits generation of active C1r (Fig. 5), which is an essential event that precedes substrate cleavage by C1s (Fig. 1*A*). As SALO blocks an earlier event within the CP than sutimlimab, it is possible that SALO could hold certain therapeutic advantages insofar as C1 inhibition is concerned (Fig. 1*A*). Unfortunately, not much is known about the antigenicity of SALO in human populations. Nevertheless, its potential utility would seem to place a premium on identifying any molecules that mimic the SALO mechanism for inhibiting C1r activation.

Zymogen activation is a seemingly ubiquitous process used to regulate the catalytic properties of enzymes in bacteria, fungi, plants, and animals. Whereas some of the best understood examples have come from classical structure–function studies on digestive proteases like trypsin(ogen) and chymotrypsin(ogen) (31), cascades of protease zymogen activation also form the basis of biochemical networks that serve broad roles in defense and repair. This includes not only the complement (1, 2, 3) and coagulation systems (32) in humans and other mammals but conceptually related pathways that fulfill analogous roles in various insect species, such as *Anopheles gambiae*, *Drosphila melanogaster*, and *Manduca sexta*, among others (33). Despite myriad studies on such systems across the last several decades, we are unaware of any endogenous regulators or pathogen/parasite-derived inhibitory molecules that interfere with zymogen activation by shielding a scissile bond from cleavage, as does SALO. Considering the numerous zymogens present in blood, as well as the remarkable diversity among hematophagous organisms and blood-borne pathogens, we suspect that additional inhibitors like SALO exist in nature and remain to be discovered.

## Experimental procedures

### Protein samples

Native C1 complex, C1r proenzyme, C1r enzyme, C1s proenzyme, C1s enzyme, and C4 purified from human serum were obtained from Complement Technologies.

For prokaryotic expression, DNA encoding WT SALO was subcloned into the pT7HMT vector and transformed into *E. coli* BL21(DE3) cells (34, 35). Cultures were grown to an absorbance of 0.6 to 0.8 at 600 nm, induced with 1 mM IPTG, and incubated for 16 h at 37 °C. Cells were harvested, lysed in 100 mM Tris–HCl (pH 8.0), 10 mM imidazole, and 6 M guanidine HCl, and clarified by centrifugation. Recombinant SALO proteins were purified using Ni–NTA affinity chromatography under denaturing conditions. Eluted proteins were reduced with 1 mM Tris(2-carboxyethyl)phosphine hydrochloride at 37 °C for 30 min and then dialyzed overnight against 2 l of 100 mM Tris–HCl (pH 8.6), 20 mM glycine, 2 mM l-cysteine, 0.5 mM EDTA, and 2.5 M urea. Each sample was then dialyzed overnight against 4 l of PBS (pH 7.4). The affinity tag was removed by incubating with recombinant tobacco etch virus protease at a 100:1 mass ratio overnight at room temperature. The sample was then applied to a 5-ml HisTrap HP column using AKTA FPLC (Cytiva), and the unbound fraction was collected thereafter. The protein was further purified on a Superdex 75 26/60 column equilibrated in Hepes-buffered saline (HBS; pH 7.4). Purified proteins were analyzed by SDS-PAGE, pooled, and stored at 4 °C or −80 °C.

For eukaryotic expression, DNAs encoding recombinant SALO proteins and zC1r-12SP were synthesized with a C-terminal octahistidine tag and subcloned into the pcDNA3.1+ vector (GeneScript). Large-scale plasmid preparations were performed using a QIAGEN Plasmid Maxi kit. Transient transfections into HEK293(T) cells (American Type Culture Collection) were achieved with polyethyleneimine MAX (36, 37). After collecting 1 l of the conditioned medium, it was concentrated to 150 ml *via* tangential flow filtration and buffer-exchanged into native binding buffer (20 mM Tris [pH 8.0], 20 mM imidazole, and 500 mM NaCl). The samples were then loaded onto a 5-ml HisTrap HP column using an AKTA FPLC (Cytiva) and eluted with native elution buffer (20 mM Tris [pH 8.0], 500 mM imidazole, and 500 mM NaCl). Fractions containing the target protein, as determined by SDS-PAGE, were further purified on a Superdex 75 26/60 column equilibrated in HBS (pH 7.4). Purified proteins were pooled, quantified, and stored at 4 °C or −80 °C for later use.

### CD spectropolarimetry

The structural integrity of various SALO proteins was assessed using CD spectropolarimetry. Experiments were performed on a Jasco J-815 CD spectrophotometer with a 1-mm pathlength jacketed cylindrical quartz cuvette. Proteins were dissolved in 5 mM Hepes (pH 7.4), 35 mM NaCl, and concentrations were determined by absorbance at 280 nm using a DeNovix DS-11 spectrophotometer. CD measurements were conducted at a final protein concentration of 37.5 μM. Spectra were recorded over a wavelength range of 260–190 nm at a scan rate of 50 nm/min with a step interval of 1 nm. For each sample, an average of five scans was used to generate a composite spectrum, followed by subtraction of a blank measurement. The resulting spectra were smoothed using the Savitzky–Golay algorithm provided in the Spectra Analysis software (Jasco, Inc).

### Mass spectrometry

The intact mass of purified SALO proteins was determined using LC–MS. The protein sample, at an approximate concentration of 1.0 mg/ml, was filtered through a 0.2 μm PTFE centrifugal filter (Millipore) prior to analysis. The filtered sample was introduced into a Waters LC–MS system, where it underwent initial desalting on a trapping column (ACQUITY UPLC M-Class Symmetry C_18_ Trap Column, 100 Å, 5 μm, 300 μm × 50 mm; Waters). Separation was on an analytical column (ACQUITY UPLC M-Class BEH C_4_ Column, 1.7 μm, 300 μm × 150 mm; Waters) under a gradient elution profile. Mobile phase A consisted of 0.1% (v/v) formic acid in water, and mobile phase B consisted of 0.1% (v/v) formic acid in acetonitrile. The gradient ranged from 15% (v/v) to 60% (v/v) mobile phase B over a duration of 20 min. MS was performed in positive and MSe mode with the capillary voltage set to 3.0 kV and the cone voltage set to 100 V. All data processing was conducted using Waters MassLynx software. Two biological and two technical replicates were performed on the eSALO-WT sample to ensure consistency of the measurements.

### SPR binding studies

SPR experiments were performed on a BiaCore T-200 instrument at 25 °C using HBS-T (20 mM Hepes [pH 7.4], 140 mM NaCl, and 0.005% [v/v] Tween-20) as the running buffer with a flow rate of 30 μl/min.

Initial SPR screening experiments were performed by immobilizing eSALO-WT on a CMD200M SensorChip (Xantec Bioanalytics). Standard *N*-hydroxysuccinimide/ethyl-*N*′-(3-(dimethylamino)propyl)carbodiimide activation was used to facilitate amine coupling to the surface (38). The experimental flow cell (fc2) had a final immobilization level of 720 RU eSALO-WT, whereas an ethanolamine-quenched flow cell (fc1) was used as a reference. Serial two-fold dilutions (15.6–1000 nM) of C1 complex, C4, C1r proenzyme, C1r enzyme, C1s proenzyme, and C1s enzyme were injected over the sensor chip in reference subtraction mode; additional experiments were performed for C1 complex and C1r proenzyme using a lower concentration dilution series (1.56–100 nM). Each injection included a 90 s association phase followed by a 180 s dissociation phase. Surface regeneration was achieved by a 60 s pulse of 20 mM glycine (pH 2.2) and 1.5 M NaCl. Reference-subtracted sensorgrams were processed using Biacore T-200 Evaluation Software, version 3.2.1 (Cytiva Life Sciences). In this initial series of experiments, sensorgrams were analyzed using a 1:1 binding model to derive an apparent *K*_*D*_ and estimates of the association and dissociation rate constants.

To compare binding of eSALO-WT to C1r proenzyme and zC1r-12SP, a low immobilization level surface was prepared using capture of biotinylated eSALO-WT. Biotinylation was achieved by mixing EZ-Link *N*-hydroxysuccinimidyl-PEG4-Biotin (Thermo Fisher Scientific) with eSALO-WT in a 2:1 M ratio according to the manufacturer’s suggestions. After a 30 min incubation at room temperature, the reaction was terminated by adding 50 mM Tris (pH 8.0). Streptavidin was immobilized on two flow cells of a CMD200M chip (Xantec Bioanalytics) *via* the amine-coupling method described previously (38). The reference surface (fc1) was prepared by injecting biotin (10 nM) three times, whereas the experimental surface (fc2) was prepared by injecting biotinylated eSALO-WT (50 nM) to achieve an immobilization level of 97 RU. Single-cycle kinetic analysis was performed using five concentrations of C1r proenzyme (25, 50, 100, 200, and 400 nM) and zC1r-12SP (6.25, 12.5, 25, 50, and 100 nM). Surface regeneration between experimental cycles was achieved using a single pulse of 20 mM glycine (pH 2.2) and 1.5 M NaCl. Reference-subtracted sensorgrams were analyzed using BiaCore T-200 evaluation software v3.2.1 (Cytiva Life Sciences).

An analogous ligand-capture protocol was used to compare eSALO-WT with its mutants. Two separate CMD200M sensor chips (Xantec Bioanalytics) were prepared with streptavidin immobilized on all four flow cells. For both chips, fc1 served as the reference surface after three injections of biotin (10 nM). On the first chip, biotinylated eSALO-WT was captured on fc2 (66 RU), biotinylated eSALO-Y51F was captured on fc3 (56 RU), and biotinylated eSALO-E58A was captured on fc4 (50 RU). On the second chip, biotinylated eSALO-WT was captured on fc2 (69 RU), biotinylated eSALO-DN was captured on fc3 (56 RU), and biotinylated eSALO-DNYE was captured on fc4 (73 RU). Binding of C1r proenzyme and zC1r-12SP to SALO proteins was assessed using single-cycle kinetics and the dilution series for each analyte described in the preceding paragraph. Because of improved quality of fitting to the experimental data, sensorgrams were analyzed using a two-state binding model to derive an apparent *K*_*D*_ with a mechanism that incorporates a single second-order rate constant (*i.e.*, *k*_a-1_) and three first-order rate constants (*i.e.*, *k*_a-2_, *k*_d-1_, and *k*_d-2_). Values from the fits are presented as the mean ± SD from a minimum of three technical replicates and are included in the accompanying table (Table 2).

### Crystallization, X-ray diffraction data collection, structure solution, and refinement

The complex of eSALO-WT and zC1r-12SP was prepared by mixing the individual monomers at molar ratios of 2:1, followed by purification *via* size-exclusion chromatography using a Superdex S75 26/60 column (Cytiva Life Sciences). Fractions containing the eSALO-WT–zC1r-12SP complex were identified by SDS-PAGE, buffer exchanged into 10 mM Hepes (pH 7.5), 50 mM NaCl, and concentrated to 1.5 mg/ml. Initial crystallization conditions were determined through vapor diffusion of sitting drops using a Crystal Gryphon liquid handling system (Art Robbins Instruments, LLC) along with the Index HT and PEGRx HT screening kits (Hampton Research Corp). Individual crystals for X-ray diffraction studies were grown through vapor diffusion of hanging drops at 20 °C by mixing 1 μl of sample with 1 μl of precipitant solution (0.2 M ammonium sulfate, 0.1 M Bis–Tris [pH 5.5], and 20% [w/v] polyethylene glycol 3350) and equilibrating over 500 μl of precipitant solution. Rod-like crystals appeared within 2 days and grew to their final size over the course of 2 weeks. Crystals were cryopreserved after a short incubation in precipitation buffer containing an additional 15% (w/v) polyethylene glycol 3350, followed by flash cooling in liquid nitrogen.

The complex of eSALO-Y51F and zC1r-12SP was prepared using the same method described previously, with the exception that the sample was concentrated to 3 mg/ml. Individual crystals for X-ray diffraction studies were grown using the same general procedure as mentioned earlier but used a different precipitant solution (0.2 M ammonium citrate tribasic [pH 7.0], 20% [w/v] polyethylene glycol 3350). Crystals were cryopreserved after a short incubation in precipitation buffer containing an additional 15% (v/v) glycerol, followed by flash cooling in liquid nitrogen.

X-ray diffraction data for the eSALO-WT–zC1r-12SP complex were collected at NSLS-II beamline 19-ID of Brookhaven National Laboratory. Reflections were indexed, integrated, and scaled using HKL-2000 (39). The structure was solved by molecular replacement using PHASER (40) as implemented in the PHENIX suite (41) with the structures of SALO (PDB entry: 5KX4 (12)) and C1r-12SP (PDB entry: 2QY0 (42)) as search models. Iterative cycles of model building and reciprocal space positional, *B*-factor, and TLS refinement were carried out using COOT (43) and PHENIX.REFINE (41), respectively, without the use of noncrystallographic symmetry restraints/constraints. The final model contains two copies of the eSALO-WT–zC1r-12SP complex in the asymmetric unit. Positive Fo–Fc electron density near the side chain of Y51 in both copies of eSALO-WT was observed during model building; upon confirmation of an increased *m/z* ratio consistent with tyrosine sulfation in the eSALO-WT sample by MS, this position was modeled as sulfotyrosine X51 in both copies of eSALO-WT. Both copies of eSALO-WT in the final model consist of residues 24 to 112 (UniProtKB code: Q5WPZ4). The first molecule of zC1r-12SP (chain C) consists of residues 308 to 416, 421 to 600, and 604 to 703, whereas the second molecule (chain D) consists of residues 308 to 415, 421 to 598, and 604 to 703 (human C1r; UniProtKB code: P00736). Although these chain breaks were included because of weak electron density, the zymogen activation loop spanning residues 458 to 468, including the scissile bond, remained intact in both zC1r-12SP molecules.

X-ray diffraction data for the eSALO-Y51F–zC1r-12SP complex were collected at ALS beamline 5.0.1 at the Berkeley National Laboratory. Reflections were processed as described previously, and the structure was solved by molecular replacement using the eSALO-WT–zC1r-12SP structure as a search model. Model building and refinement followed the same general procedures described previously. The final model consists of two molecules of eSALO-Y51F–zC1r-12SP in the asymmetric unit. Both copies of eSALO-Y51F in the final model consist of residues 24 to 112 (UniProtKB code: Q5WPZ4), with the exception of the Y51F mutation. The first molecule of zC1r-12SP (chain C) consists of residues 308 to 417, 420 to 508, 515 to 597, and 605 to 703 except 629, whereas the second molecule (chain D) consists of residues 308 to 417, 423 to 507, 513 to 597, and 604 to 703 (human C1r; UniProtKB code: P00736). As with the eSALO-WT–zC1r-12SP structure, chain breaks were included because of weak electron density, but the zymogen activation loop was intact in both zC1r-12SP copies.

Refined models for eSALO-WT–zC1r-12SP and eSALO-Y51F–zC1r-12SP have been deposited in the PDB under accession codes 9EKD and 9EKE, respectively. Additional descriptive statistics for X-ray diffraction data collection and model refinement are included in the accompanying table (Table 1).

### C1r-proenzyme autoactivation assay

Inhibition of C1r-proenzyme autoactivation was assessed by detecting the cleavage of C1r-proenzyme using SDS-PAGE. All assays were established on ice in 10 μl total volume and contained 3.3 μM C1r-proenzyme and 8.3 μM SALO protein. Each trial contained 6 μl of C1r-proenzyme at 0.5 μg/μl (supplied in a buffer of 10 mM imidazole [pH 6.0], 400 mM NaCl, 1 mM CaCl_2_) and 4 μl of SALO at 0.25 μg/μl in HBS; an identical sample containing C1r-proenzyme alone incubated at 4 °C served as a negative control for cleavage, whereas a sample containing 1 μg of C1r-enzyme alone was included as a positive control for cleavage. Reaction mixtures were then incubated as appropriate for 10 h at 37 °C. Reactions were terminated by adding SDS-PAGE sample buffer containing reducing agent, followed by heating at 95 °C for 5 min. The cleavage products were separated by SDS-PAGE and visualized by Coomassie staining.

### Hemolysis assays

CP hemolysis assays were conducted in a 100 μl reaction mixture containing 0.5 μl of normal human serum (Innovative Research), 59.5 μl of 0.1% (w/v) gelatin veronal buffer (GVB++; Complement Technologies), 20 μl of SALO protein in GVB++ at the desired concentration, and 20 μl of antibody-sensitized sheep erythrocytes (3 × 10^8^/ml in GVB++). The mixture was incubated at 37 °C for 30 min, followed by centrifugation at 200*g* for 10 min. The supernatant was then diluted fourfold in water, and the absorbance was measured at 412 nm. The percentage of lysis inhibition was calculated using the formula: 100-[100∗(OD_412, sample_−OD_412, buffer_/(OD_412, water_−OD_412, buffer_)], and the IC_50_ was determined by a four-parameter fit using GraphPad Prism, version 10.1.1 (GraphPad Software, Inc).

## Data availability

The atomic coordinates and structure factors for crystal structure determinations have been deposited in the PDB (http://wwpdb.org/) and are publicly available through the accession codes provided (Table 1). All other experimental data are available upon request by contacting the corresponding author B. V. G. (geisbrechtb@ksu.edu).

## Supporting information

This article contains supporting information.

## Conflict of interest

The authors declare that they have no conflicts of interest with the contents of this article.
