# Supplementary material for: Blocking activation of the C1r zymogen defines a novel mode of complement inhibition
Source: J Biol Chem. 2025 Feb 11;301(3):108301. doi: 10.1016/j.jbc.2025.108301 (PMC11928764; doi:10.1016/j.jbc.2025.108301)
Supplement: Supporting information [file mmc1.pdf]

SUPPORTING INFORMATION FILE

Blocking Activation of the C1r Zymogen Defines a Novel Mode of Complement Inhibition

Huiquan Duan[a], Wei Wu[b], Ping Li[b],

Samuel Bouyain[c], Brandon L. Garcia[a], and Brian V. Geisbrecht[a]

Author Affiliations:

[a]Department of Biochemistry & Molecular Biophysics
Kansas State University
Manhattan, KS 66506 USA

[b]Department of Chemistry
Kansas State University
Manhattan, KS 66506 USA

[c] Division of Biological and Biomedical Systems
School of Science and Engineering
University of Missouri-Kansas City
Kansas City, MO 64110 USA

Corresponding Author:

Brian V. Geisbrecht, Ph.D.
Department of Biochemistry & Molecular Biophysics
Kansas State University
141 Chalmers Hall
1711 Claflin Road
Manhattan, KS 66506 USA
PH: 785-532-3154
E-mail: geisbrechtb@ksu.edu

Keywords:

complement system; zymogen activation; protein structure; inhibitor; immune evasion

Running title:

Inhibiting activation of the C1r zymogen

This file contains 4 supporting figures and their corresponding legends

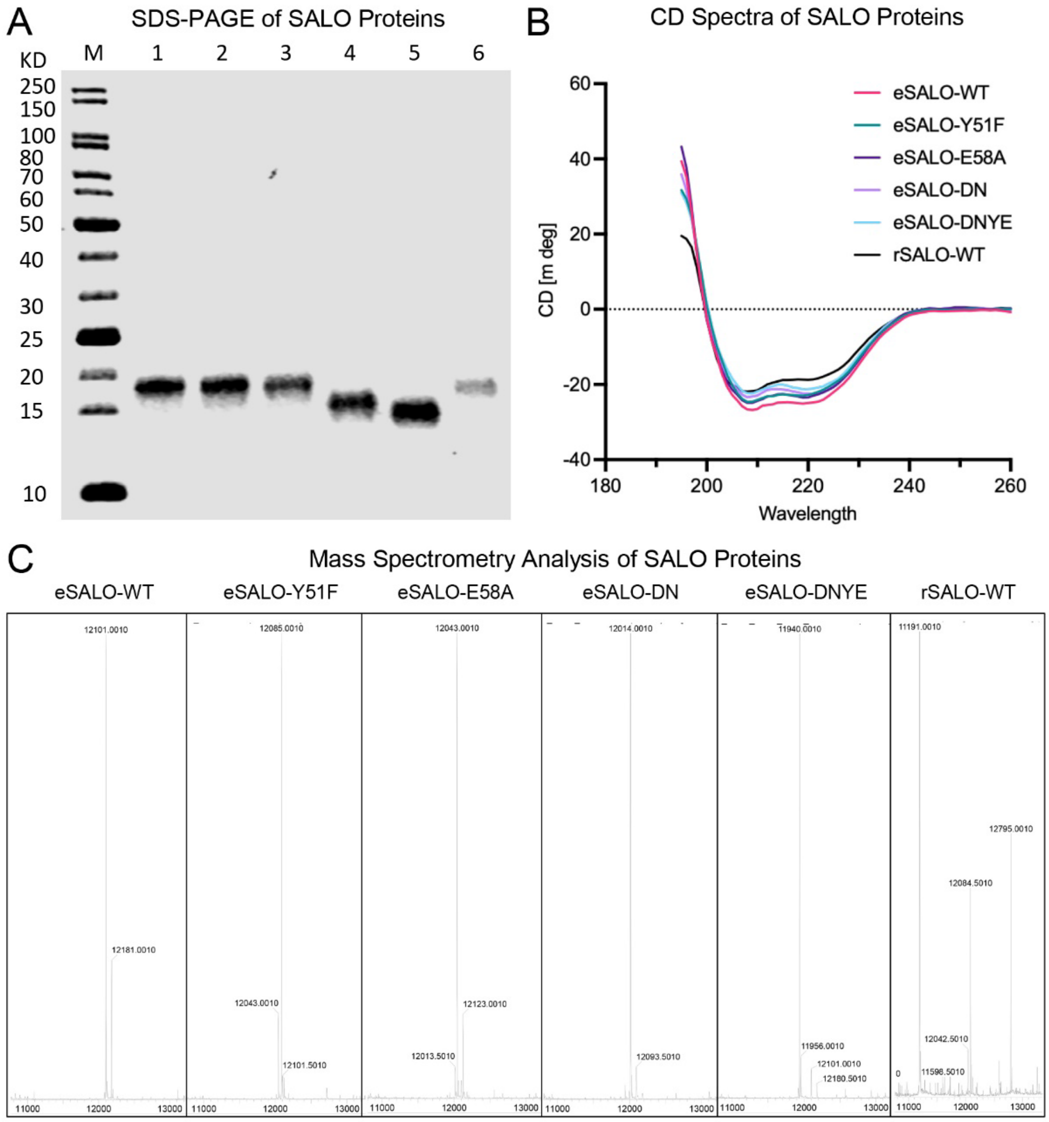

D Biochemical Parameters of SALO Proteins

Sample	Calculated M.W. (Da)	Measured M.W. (Da)	Measured M.W. with Tys (Da)
eSALO-WT	12107	12101	12181
eSALO-Y51F	12091	12085	-
eSALO-E58A	12049	12043	12123
eSALO-DN	12020	12014	12093
eSALO-DNYE	11946	11940	-
rSALO-WT	10563	11191	-

Figure S1. Supporting information pertaining the characterization of SALO proteins. (A) SDS-PAGE analysis of recombinant SALO proteins used in this study. 3 µg of each protein were processed for SDS-PAGE under reducing conditions. The identities of individual lanes are as follows: M, molecular weight markers; 1, eSALO-WT; 2, eSALO-Y51F; 3, eSALO-E58A; 4, eSALO-DN; 5, eSALO-DNYE; 6, rSALO-WT. (B) CD Spectra of recombinant SALO proteins used in this study. Spectra were collected on 37.5 µM protein dissolved in a buffer of 5 mM HEPES (pH7.4), 35 mM NaCl. A legend identifying the individual spectral traces is inset. (C) Mass spectrometry analysis of recombinant SALO proteins used in this study. Protein samples were separated by a C_4 UPLC column prior to collecting ESI-TOF data in positive and MSe mode. Regions corresponding to m/z values from 11,000 to 13,000 are presented for each SALO protein. Note the presence of an additional species at m/z ~80 units greater than the major species in all eSALO samples containing Y51, consistent with the presence of tyrosine sulfation. The major species identified also corresponds well to the expected molecular weight of each SALO protein, despite the fact that several SALO variants display anomalous migration in SDS-PAGE experiments. (D) Table of biochemical parameters for recombinant SALO proteins used in this study. Calculated molecular weights for HEK293(T)-cell expressed forms were determined from the predicted secretion signal peptide cleavage site using the SignalP 6.0 server. The difference of 6 units in m/z corresponds to loss of hydrogen atoms following formation of 3 pairs of disulfide bonds.

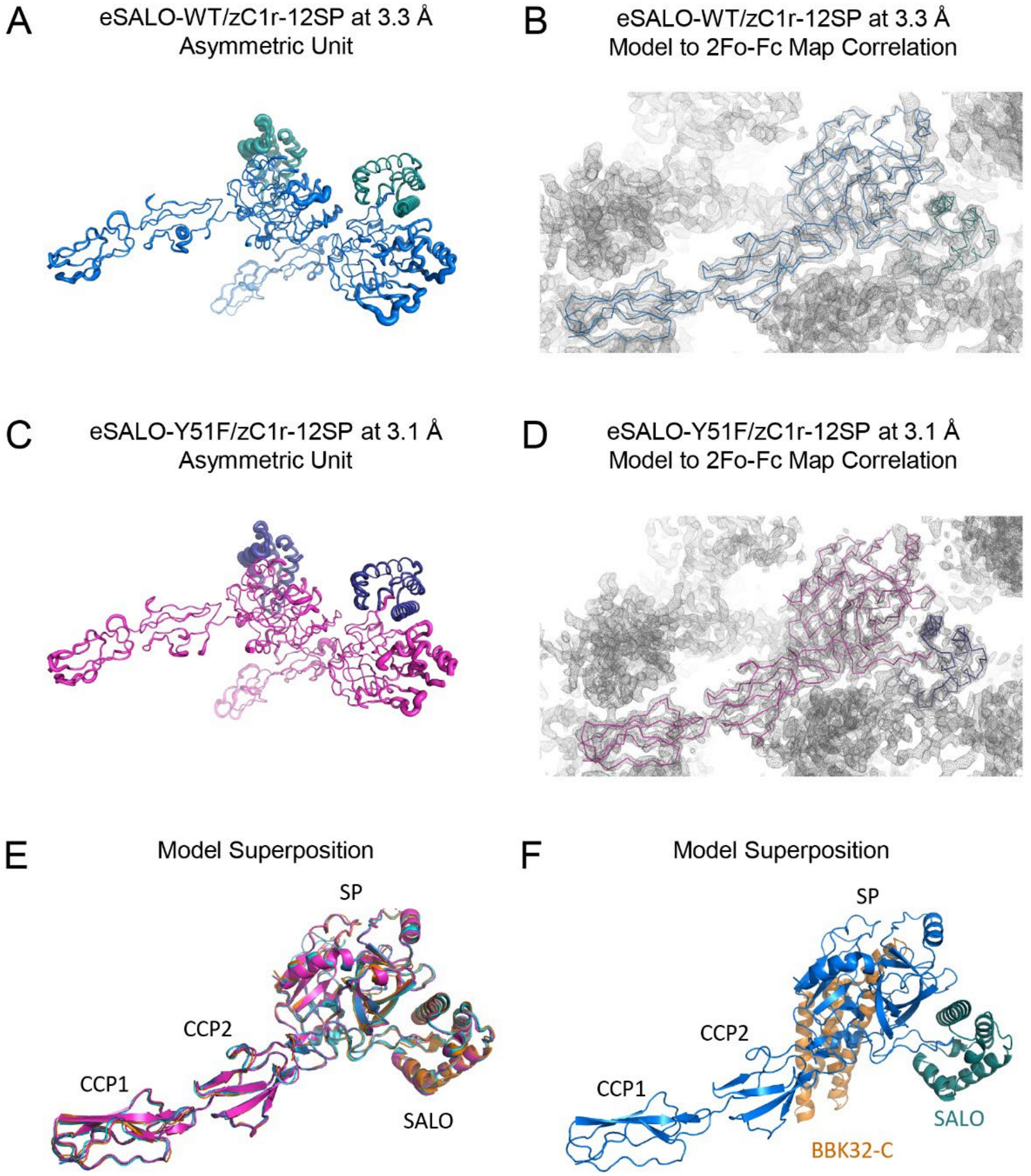

Figure S2. Supporting information pertaining to the structures of eSALO-WT and eSALO-Y51F bound to zC1r-12SP. (A) Model of the entire asymmetric unit of the eSALO-WT/zC1r-12SP crystal. eSALO-WT molecules are colored teal, while zC1r-12SP molecules are colored blue. The thickness of the tracing represents the final B-factor of that residue, with thicker tracing indicating higher B-factors. (B) Representative model to map correlation around chains A and C of the eSALO-WT/zC1r-12SP complex. The 2Fo-Fc map calculated at 3.3 Å limiting resolution and contoured at 1.2σ is drawn as a grey mesh. Proteins are colored as in Panel A. (C) Model of the entire asymmetric unit of the eSALO-Y51F/zC1r-12SP crystal. eSALO-Y51F molecules are colored purple, while zC1r-12SP molecules are colored magenta. The thickness of the tracing represents the final B-factor of that residue, with thicker tracing indicating higher B-factors. (D) Representative model to map correlation around chains A and C of the eSALO-Y51F/zC1r-12SP complex. The 2Fo-Fc map calculated at 3.1 Å limiting resolution and contoured at 1.2σ is drawn as a grey mesh. Proteins are colored as in Panel C. (E) Superposition of complexes from the eSALO-WT/zC1r-12SP and eSALO-Y51F/zC1r-12SP structures. The two copies of the eSALO-WT/zC1r-12SP structure are colored in blue and cyan, respectively, while the two copies of the eSALO-Y51F/zC1r-12SP structure are colored in magenta and orange, respectively. (F) Comparison of the binding sites for SALO and *B. burgdorferi* BBK32-C. SALO is colored teal, while BBK32-C is colored light orange. The BBK32-C structure is taken from PDB entry 7MZT.

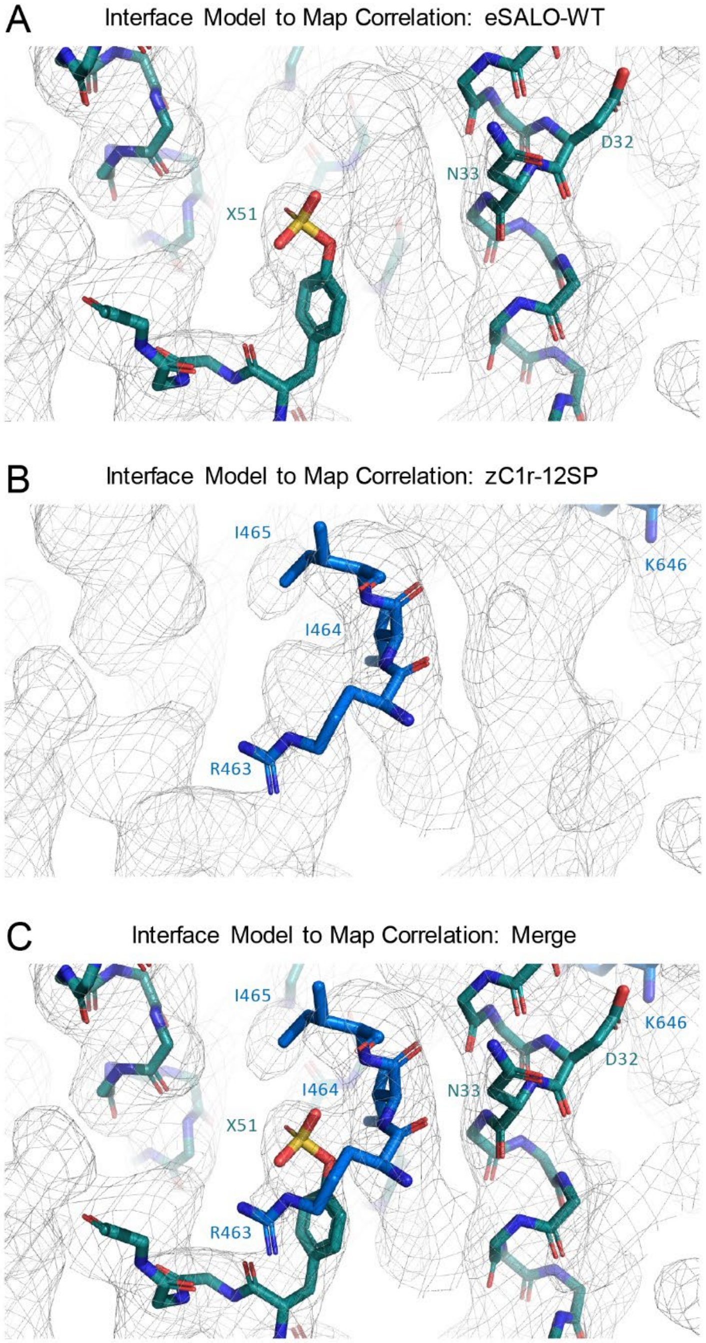

Figure S3. Supporting information pertaining to the interface of eSALO-WT/zC1r-12SP. (A) Representative model to map correlation at the interface of the eSALO-WT/zC1r-12SP complex represented by chains A and C in the PDB entry. The 2Fo-Fc map calculated at 3.3 Å limiting resolution and contoured at 1.2σ is drawn as a grey mesh, while eSALO-WT residues near the interface are drawn with their carbon atoms in teal. Sidechains of residues highlighted in Fig. 3 are shown in ball and stick convention. Note the central position of the sulfated tyrosine-51, X51. (B) Representative model to map correlation at the interface of the eSALO-WT/zC1r-12SP complex represented by chains A and C in the PDB entry. The 2Fo-Fc map calculated at 3.3 Å limiting resolution and contoured at 1.2σ is drawn as a grey mesh, while zC1r-12SP residues near the interface are drawn with their carbon atoms in blue. Sidechains of residues highlighted in Fig. 3 are shown in ball and stick convention. (C) A merged image of Panels A and B. Proteins are colored as described in the previous panels.

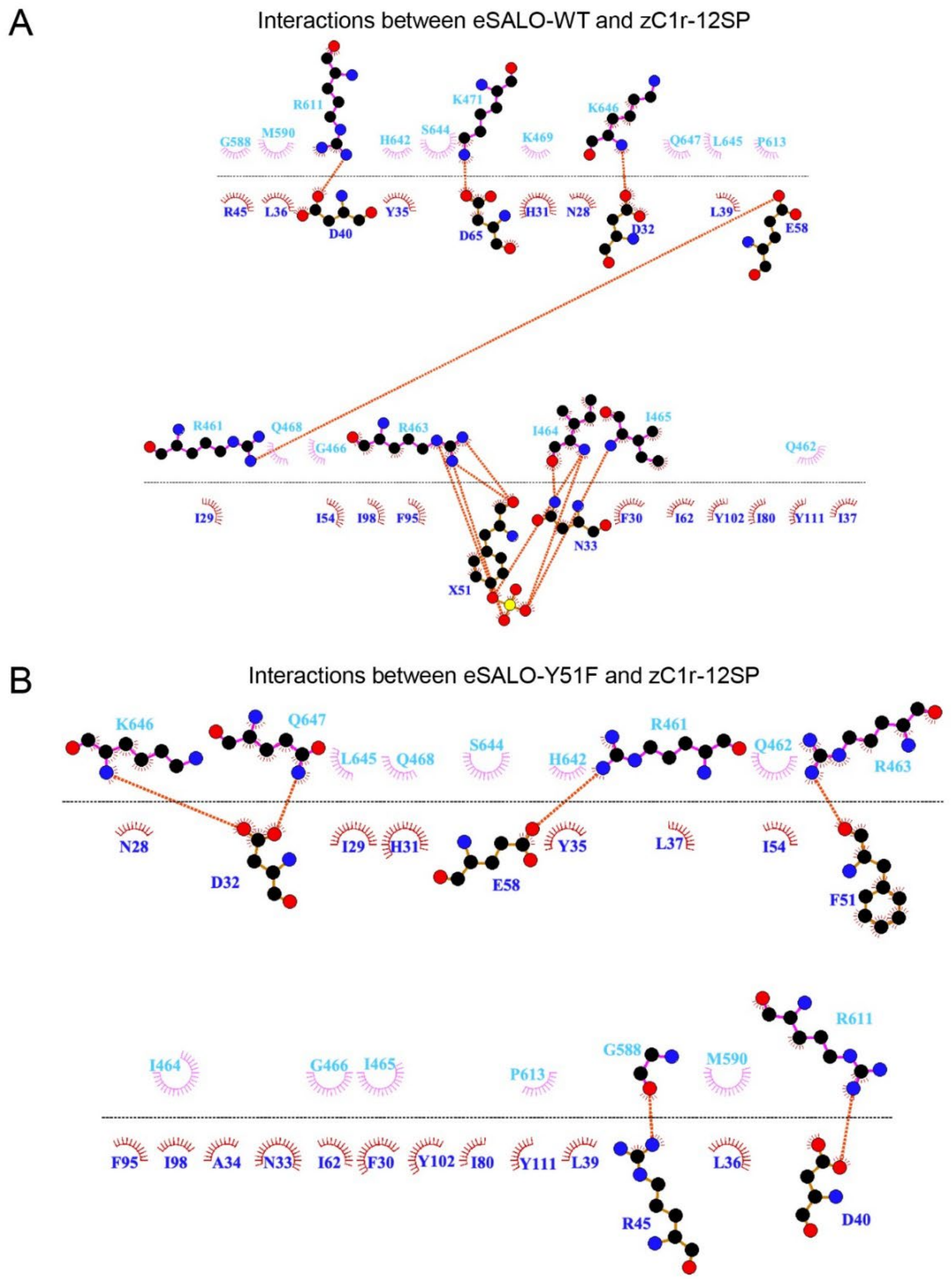

Figure S4. Further supporting information pertaining to the interfaces of eSALO-WT/zC1r-12SP and eSALO-Y51F/zC1r-12SP. (A) Schematic representation of non-covalent interactions found at the eSALO-WT/zC1r-12SP interface. Hydrogen bonds are represented by dashed orange lines, while the toothed arcs represent packing/hydrophobic interactions. Residues derived from eSALO-WT are indicated with blue typeface and appear below the dashed black line, while residues derived from zC1r-12SP are indicated with cyan typeface and appear above the dashed black line. Note the sulfate group present on eSALO-WT residue X51. (B) Schematic representation of non-covalent interactions found at the eSALO-Y51F/zC1r-12SP interface. Hydrogen bonds are represented by dashed orange lines, while the toothed arcs represent packing/hydrophobic interactions. Residues derived from eSALO-Y51F are indicated with blue typeface and appear below the dashed black line, while residues derived from zC1r-12SP are indicated with cyan typeface and appear above the dashed black line.
